# Livestock farmers’ knowledge, attitudes and practices relating to zoonoses in the Coastal Savannah zone of Ghana

**DOI:** 10.1093/inthealth/ihaf101

**Published:** 2025-09-17

**Authors:** Sylvia Afriyie Squire, Godwin Yao Ameleke, Edmund Tei Sottie, Hilda Ohene-Asa, Nancy Mensah, Dorothy Takyiakwaa

**Affiliations:** Animal Health Division, Council for Scientific and Industrial Research–Animal Research Institute, P. O. Box AH 20, Achimota, Accra, Ghana; Department of Natural Resources Management, CSIR College of Science and Technology, Ghana; Department of Natural Resources Management, CSIR College of Science and Technology, Ghana; Livelihood and Innovation Division, Council for Scientific and Industrial Research–Animal Research Institute, P. O. Box AH 20, Achimota, Accra, Ghana; Department of Natural Resources Management, CSIR College of Science and Technology, Ghana; New Products and Food Safety Division, Council for Scientific and Industrial Research–Animal Research Institute, P. O. Box AH 20, Achimota, Accra, Ghana; Animal Health Division, Council for Scientific and Industrial Research–Animal Research Institute, P. O. Box AH 20, Achimota, Accra, Ghana; Kasapreko PLC, DTD #64, Off Spintex Road Baatsonaa, Accra, Ghana; African Studies Program, Pennsylvania State University, State College, Pennsylvania, USA

**Keywords:** attitudes and practices, Ghana, knowledge, livestock farmers, socio-economic and farm characteristics, zoonoses

## Abstract

**Background:**

Zoonoses pose a significant health challenge globally, with livestock playing a major role in disease transmission. Understanding livestock farmers’ knowledge, attitudes and practices (KAP) is crucial for prevention and control.

**Methods:**

A cross-sectional survey was conducted among 150 livestock farmers in Ghana's Coastal Savannah agroecological zone. Odds ratios (ORs) and 95% confidence intervals (CIs) from logistic regression analysis assessed the association between farmers’ KAP and socio-economic and farm characteristics.

**Results:**

While 66.0% of farmers had satisfactory knowledge of specific zoonoses and 74.0% perceived preventive practices as protective, only 49.0% perceived the diseases as zoonotic and 39.3% practiced prevention satisfactorily. Farmers with fewer disease encounters (adjusted OR [aOR] 0.499 [95% CI 0.347 to 0.717]), males (aOR 3.238 [95% CI 1.213 to 8.648]) and intensive farmers (aOR 5.396 [95% CI 1.276 to 22.808]) have higher odds of having satisfactory knowledge. Conversely, males (aOR 0.261 [95% CI 0.077 to 0.885]) and farmers with more disease encounters (aOR 0.664 [95% CI 0.490 to 0.898]) have lower odds of having satisfactory perception.

**Conclusions:**

This study highlights a knowledge–practice gap among livestock farmers regarding zoonoses, influenced by factors like gender, production systems and disease encounters. Targeted training is recommended for women, extensive systems and disease-prone farms.

## Introduction

Zoonotic diseases, which can be transmitted between animals and humans, pose a substantial global public health challenge, with >200 known zoonoses.^1^ Livestock plays a crucial role in the transmission of many zoonotic diseases.^2,3^ Research has indicated that farmers exhibit diverse levels of knowledge and attitudes toward zoonotic diseases and frequently engage in suboptimal practices that are influenced by several factors.

Studies across different regions highlight the complexity of this issue. In Africa, for instance, a study in Rwanda found that despite 50.3% of smallholder livestock farmers being aware of zoonoses, risky attitudes and practices persisted, including failure to isolate sick animals.^4^ Similarly, research in Nigeria revealed significant knowledge–practice gaps among livestock farmers regarding specific zoonoses like anthrax and zoonotic tuberculosis.^5,6^ In Asia, a study in Malaysia revealed a discrepancy between ruminant farmers’ understanding of zoonotic diseases and their actual practices, with only 12% prioritising essential preventive measures like milk pasteurization despite recognizing disease transmission risks.^7^ Likewise, in Nepal, 72.1% of farmers were aware of zoonotic diseases, but only a third recognized the transmission of rabies to cattle and buffalo and none knew of leptospirosis.^8^ In Europe, a study in Greece found that factors such as education and veterinary supervision played a role in shaping farmers’ knowledge and practices.^9^

In Ghana, livestock farming has been linked to various zoonotic diseases, including anthrax, brucellosis, cryptosporidiosis and bovine tuberculosis.^10–16^ Farmers’ knowledge, attitudes and practices (KAP) play a critical role in zoonotic disease transmission, emphasising the need for a comprehensive understanding of their KAP. Research in Tamale, in northern Ghana, revealed that despite cattle farmers’ good knowledge and preventive attitudes, their practices were suboptimal, with their attitudes influenced by education and age, while their knowledge and practices were impacted by ethnicity.^17^ Another study in the Ashanti region found that education and work experience impacted poultry farmers’ knowledge of poultry diseases and zoonoses, but the factors influencing their attitudes and practices toward zoonoses were not examined statistically, although communication and economic factors were suggested as potential contributors to limited personal protective equipment (PPE) use.^18^ While these studies have advanced our understanding, significant knowledge gaps remain.

These studies were geographically limited to Ghana's Guinea Savannah zone (Tamale) and Forest zone (Ashanti Region) and focused on specific livestock species (cattle and poultry). However, Ghana's six agroecological zones exhibit significant variations in climate and vegetation that influence agricultural activities.^19^ These variations may influence the prevalence of zoonoses and farmers’ KAP. For instance, the Guinea Savannah zone features unimodal rainfall and grasslands suitable for pastoralism, whereas the Forest zone has bimodal rainfall and high poultry density.^19^ Anthrax is endemic in northern Ghana (Guinea and Sudan Savannah zones), emphasising zone-prone risk.^15^ Given these variations, similar studies on KAP related to zoonoses should be conducted in other zones and cover a broader range of livestock species for a more comprehensive understanding. Additionally, the determinants of poultry farmers’ attitudes and practices regarding zoonoses remained unclear, requiring further investigations.^18^

Thus this study investigated the KAP of a wide range of livestock farmers across three districts in the Coastal Savannah zone of Ghana, where research on KAP regarding zoonoses is limited. Specifically, it sought to describe livestock farmers’ KAP related to zoonosis, assess the influence of socio-economic and farm characteristics on their KAP and explore the underlying reasons for their KAP. Understanding the KAP of farmers is essential for controlling zoonotic diseases, as their perceptions and behaviours significantly influence disease transmission and prevention.

## Methods

### Study design

A cross-sectional survey was conducted from January to June 2022 by using an interviewer-administered questionnaire to assess the KAP among livestock farmers regarding zoonotic diseases.

### Study area

The study was conducted in the Coastal Savannah agroecological zone, located in southern Ghana along the coastal belt, where a notable knowledge gap regarding livestock farmers’ KAP towards zoonoses remained. This zone is characterised by a bimodal rainfall pattern and diverse vegetation, including shrub lands, grasslands and coastal lands, making it suitable for livestock rearing.^19^ Furthermore, the Greater Accra Region, which is part of the Coastal Savannah, has a high concentration of commercial poultry farms. Based on a convenient multistage sampling method, three districts with high livestock concentrations were purposively selected with the assistance of Ministry of Food and Agriculture (MoFA) officers. These were the Shai Osudoku and Ada West districts in the Greater Accra Region, and North Tongu district in the Volta Region. Within each district, communities with high livestock densities were purposively selected for the study.

### Study participants

A total of 150 farmers ≥18 y of age, raising at least one category of livestock (cattle, sheep, goats, pigs, rabbits, grasscutters or poultry), were invited to participate in a questionnaire survey. Inclusion criteria were having a minimum of 10 livestock and a farm located within the study area.

The sample size was determined using EpiTools, assuming a livestock farmer population of 10 000, with 8% precision, a 95% confidence interval (CI), 80% power and an expected proportion of 50% reported by a similar study in Ghana by Ziblim et al.^17^ The calculation followed the approach described previously.^20^ Given the lack of current data on the population of livestock farmers, estimates were derived from the 2010 Population and Housing Census reports, which listed 7096, 6689 and 3470 livestock keepers in Ada West, North Tongu and Shai Osudoku districts, respectively.^21–23^ Considering these figures included farmers raising various livestock species beyond the scope of this study and potential fluctuations due to urbanization, the assumption of 10000 livestock farmers was deemed reasonable.

Using a convenient sampling approach, 150 farmers were selected and interviewed, consisting of 50 from Ada West, 61 from North Tongu and 39 from Shai Osudoku districts, based on the specified inclusion criteria and their willingness to participate.

### The survey instruments

A structured questionnaire was developed to gather comprehensive information on farmers’ demographic and socio-economic status, KAP related to zoonoses and husbandry and biosecurity practices. The demographic and socio-economic section captured data on farmers’ age, education, occupation, livestock species and numbers, experience, farm location, training and workforce size. The knowledge section assessed farmers’ understanding of zoonotic disease transmission (between livestock and humans, humans to livestock and livestock to livestock or other animals) and awareness of specific diseases (avian influenza or bird flu, rabies, swine flu, anthrax, tuberculosis, brucellosis, diarrhoea, worm infestation, skin diseases and ectoparasites). The perception section explored farmers’ views on disease transmissibility to humans, while the attitude section evaluated their perceptions of protective measures, including PPE use and sanitary practices (e.g. wearing masks, gloves and safety boots; handwashing; and changing clothes). The practices section investigated farmers’ self-reported adherence to these protective measures, providing insights into the gap between knowledge, attitudes and practices.

### Data collection

The survey questionnaire was administered face to face, either in English or the respondent's local language, with the assistance of an interpreter when necessary. Visual aids, including pictures of various disease symptoms, were used to facilitate the understanding and explanation of diseases. The survey instrument was pretested and validated by selected farmers, experts and the ethics committee before administration. All data were collected according to the approved ethical protocol.

### Data management and analysis

Collected survey data were exported from KoboToolbox (https://www.kobotoolbox.org) to Excel (Microsoft, Redmond, WA, USA), where unique identification codes were assigned to ensure participant anonymity and confidentiality.

Quantitative data were analysed using SPSS 25 for Windows (IBM, Armonk, NY, USA). Descriptive statistics summarized farmers’ socio-economic characteristics, knowledge, perceptions, attitudes and practices. Logistic regression assessed associations between these factors and socio-economic and farm characteristics and the Nagelkerke R^2^ was used to evaluate model fit.

The socio-economic characteristics of farmers were analysed by computing the proportions of farmers belonging to various categories. The proportions of farmers with knowledge about zoonotic disease transmission (livestock to human, human to livestock and between livestock) were calculated, with farmers’ knowledge measured on a scale of 0–2. The proportions of farmers aware of specific diseases and their transmissibility to humans were computed and illustrated with bar charts.

The proportions of farmers who perceived PPE and sanitary practices as protective against livestock diseases were computed and illustrated. Practice scores were also assessed, evaluating the use of PPE and engagement in sanitary practices. Overall KAP scores were analysed using descriptive statistics (mean, standard deviation [SD], minimum and maximum), with scores calculated based on 10 items each. For knowledge and attitude questions, a ‘yes’ response received a score of 2, ‘no idea’ received a score of 1 and ‘no’ received a score of 0. For practice questions, a ‘yes’ response received a score of 2, ‘needs improvement’ received a score of 1 and ‘no’ received a score of 0, resulting in a maximum overall score of 20 and a minimum score of 0.

The association between KAP related to zoonotic diseases and socio-economic and farm characteristics was assessed using adjusted odds ratios (aORs) obtained from binary logistic regression. The dependent variable, overall KAP score, was dichotomized as poor (below average score) or satisfactory (equal to or above average score). The independent variables included sex (male or female), production system (extensive or intensive), farm size (in tropical livestock units), years of livestock farming experience and number of diseases encountered on the farm.

## Results

### Socio-economic characteristics

Table 1 presents the socio-economic characteristics of farmers surveyed. The majority of the farmers (79%) were male and 30–50 y of age (42%) or >50 y of age (42%). Only 9% of the farmers had a tertiary education, but more than half of them (52%) had >10 y of livestock farming experience. Most of the farmers (93%) kept livestock alongside crop farming and/or other enterprises, with 87% owning their farms and 84% raising multiple livestock types including ruminants, poultry, pigs and/or rabbits. The majority of farmers (57%) also kept pets. Notably, 72% of farmers lacked formal training in livestock production. The farms were predominantly small-scale, with fewer than five workers, and a substantial portion of farm workers (37%) were <15 y of age.

**Table 1.** Socio-economic characteristics of livestock farmers.

Characteristic	Category	n (%)
Region where farm is located	Greater Accra	89 (59.3)
	Volta	61 (40.7)
District where farm is located	Ada West	50 (33.3)
	North Tongu	61 (40.7)
	Shai Osudoku	39 (26.0)
Sex	Male	118 (78.7)
	Female	32 (21.3)
Age group (years)	18–29	23 (15.3)
	30–50	63 (42.0)
	≥50	64 (42.7)
Level of education	No formal education	34 (22.7)
	Basic (primary/junior high school)	50 (33.3)
	Secondary	53 (35.3)
	Tertiary	13 (8.7)
Years in livestock farming	<1	4 (2.7)
	1–5	35 (23.3)
	5–10	33 (22.0)
	>10	78 (52.0)
Category of farmer	Livestock only	11 (7.3)
	Livestock and crop farmer	77 (51.3)
	Livestock and others	41 (27.3)
	Livestock, crop farmer and other	21 (14.0)
Type of livestock kept	Ruminant	28 (18.7)
	Ruminant, pig and poultry	8 (5.3)
	Ruminant and pig	3 (2.0)
	Ruminant and poultry	95 (63.3)
	Pig	2 (1.3)
	Pig and poultry	3 (2.0)
	Poultry	10 (6.7)
	Ruminants, poultry and rabbit	1 (0.7)
Do you keep a pet?	No	64 (42.7)
	Yes, dog and/or cat	86 (57.3)
Do you own the farm or part?	Yes	131 (87.3)
	No, I am Family Member	15 (10.0)
	No, I am an Employee	4 (2.7)
Any formal training in livestock farming?	Yes	41 (27.3)
	No	109 (72.7)
If you had formal training in livestock farming, when was the latest? (years ago)	<1	4 (9.8)
	1–5	17 (41.5)
	6–10	7 (17.1)
	>10	6 (14.6)
	Not sure	7 (17.1)
Number of farm workers	<5	90 (60.0)
	≥5	60 (40.0)
Workers under 15 years?	Yes	56 (37.3)
	No	94 (62.7)

Ruminants: cattle, sheep and goats, with goats being the most common at 75%; poultry: exotic/commercial chickens, local chickens, ducks, guinea fowls, turkeys and doves, with local chickens accounting for 75%.

### Farmers’ knowledge in relation to zoonoses

**Figure 1.**
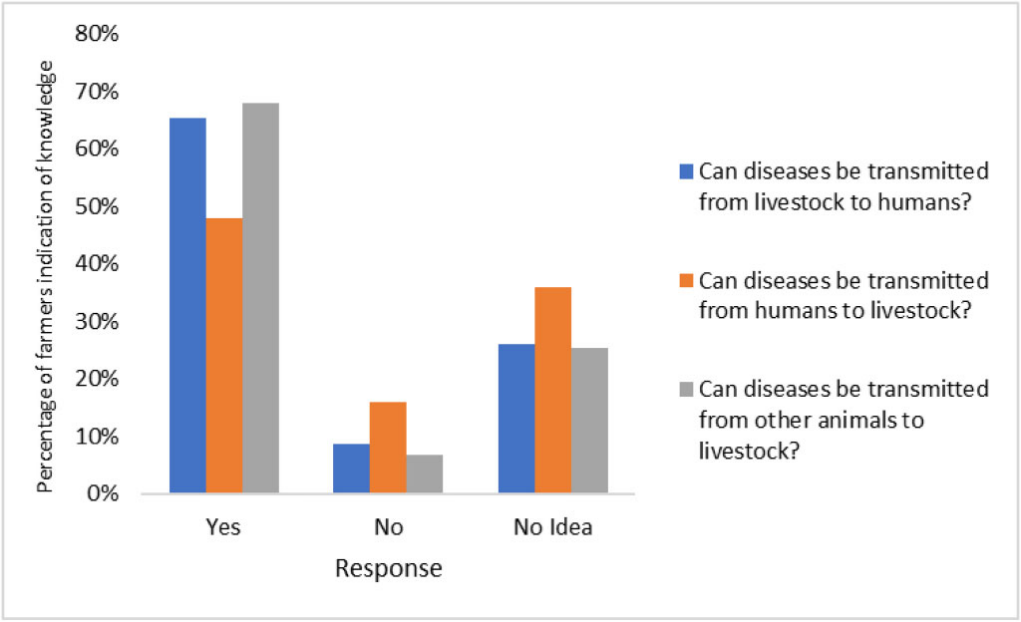
Proportions of farmers with knowledge about livestock-to-human, human-to-livestock and livestock-to-other animal transmission of diseases.

Most farmers acknowledged disease transmission between humans, livestock and other animals, with 65% recognizing livestock-to-human transmission, 48% recognizing human-to-livestock transmission and 68% recognizing transmission between livestock and other animals (Figure 1).

**Figure 2.**
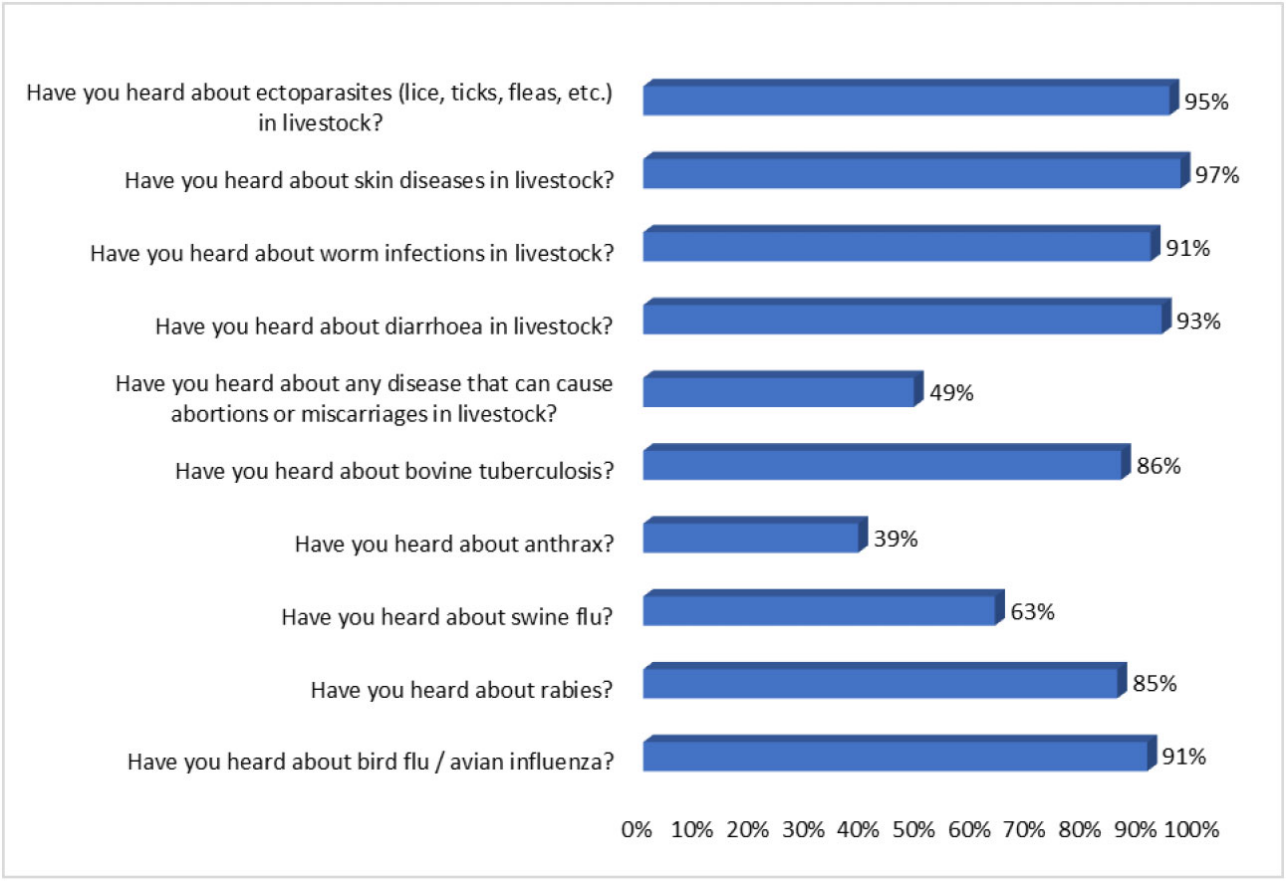
Proportion of farmers who were aware of specific zoonotic diseases.

Most farmers (>70%) were aware of 8 of 10 diseases or parasites, including bird flu, rabies, swine flu, tuberculosis, diarrhoea, skin diseases, worms and ectoparasites. While about 90% of farmers knew about bird flu, diarrhoea, worm infections, skin diseases and ectoparasite infestations, less than half were familiar with abortion-causing diseases and anthrax, with anthrax being the least recognized (Figure 2).

### Farmers’ perceptions and attitudes in relation to zoonoses

**Figure 3.**
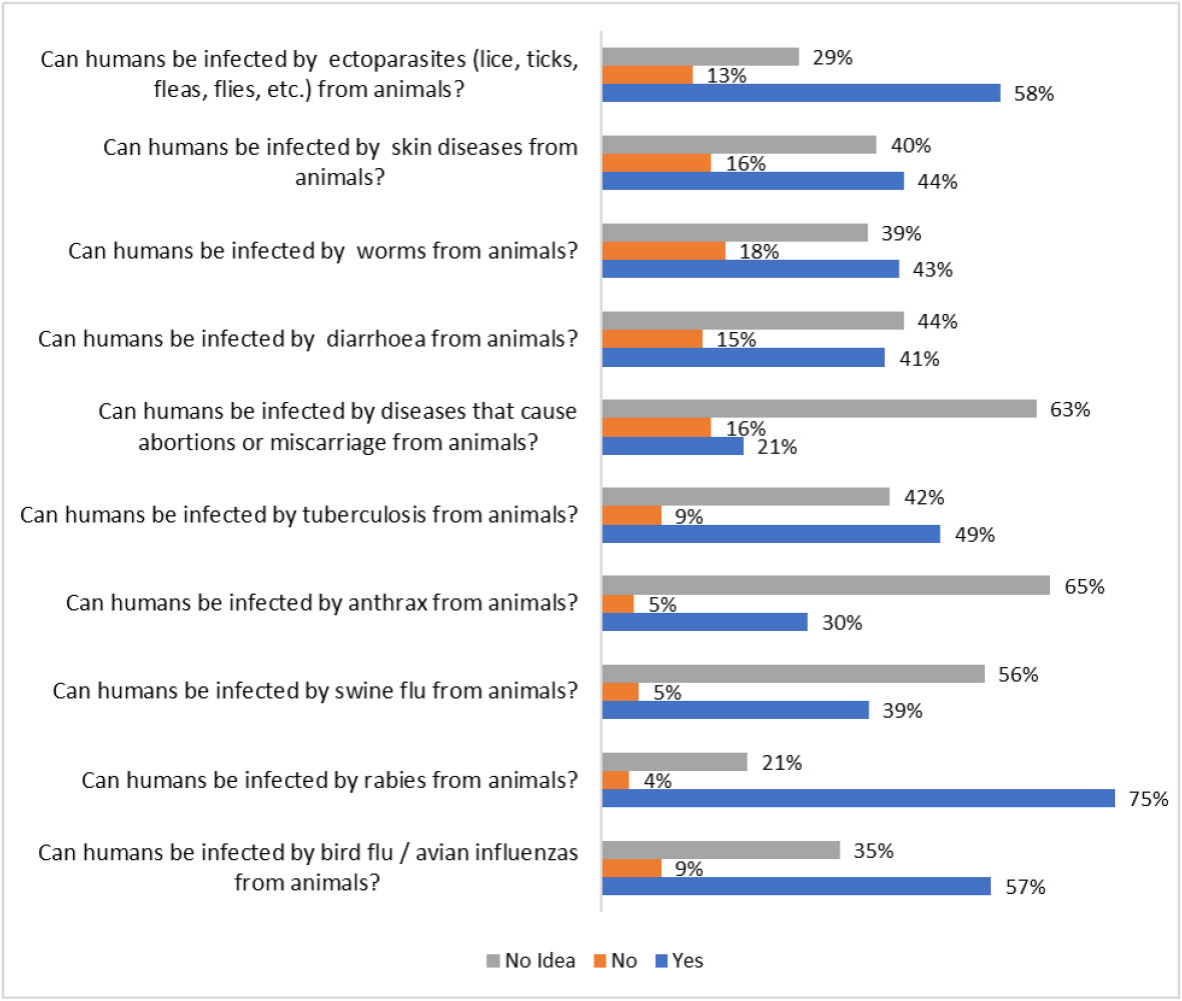
Proportion of farmers perceiving animal diseases as transmissible to humans.

Figure 3 presents the varied perceptions among farmers regarding the zoonotic potential of specific animal diseases. Most farmers recognized ectoparasites, rabies and bird flu as transmissible to humans. Conversely, uncertainty prevailed regarding swine flu, anthrax and abortion-causing diseases, with most farmers lacking knowledge on their zoonotic potential. Notably, <18% of farmers believed that any of the diseases were not transmissible to humans. Rabies was perceived as the most likely to be transmitted to humans, while anthrax was considered the least transmissible.

**Figure 4.**
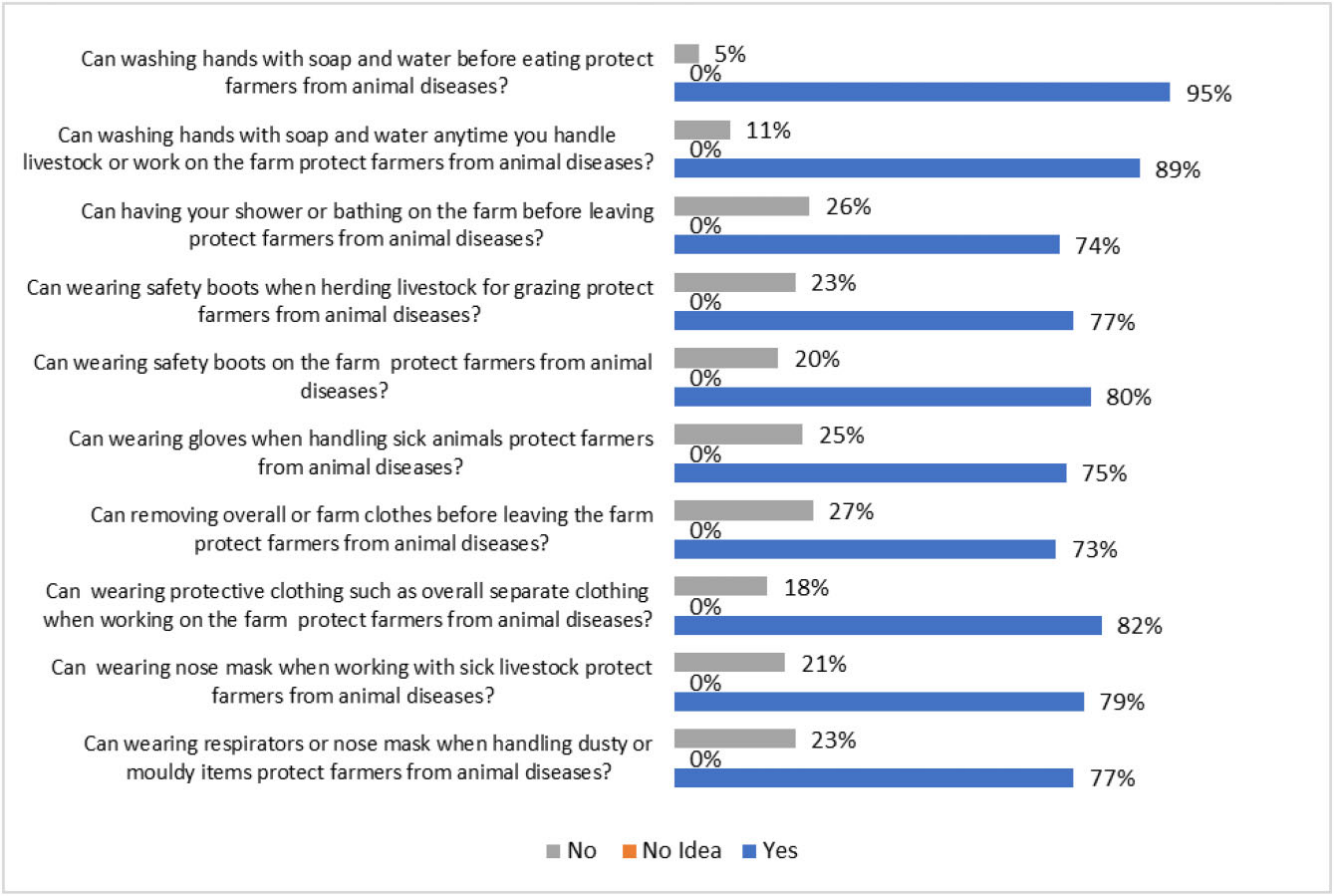
Farmers’ perception of protection offered by PPE and sanitary practices.

Figure 4 illustrates farmers’ perceptions regarding the protective value of PPE and sanitary practices. A significant majority (75–95%) of farmers agreed that each of the 10 practices presented offered protection. Markedly, no farmer believed that these practices offered no protection. However, a proportion of farmers (5–27%) was uncertain about the protective benefits. The practices most widely recognized as protective were washing hands with soap before eating (95%) and after handling livestock (89%).

### Farmers’ practices in relation to zoonoses

**Figure 5.**
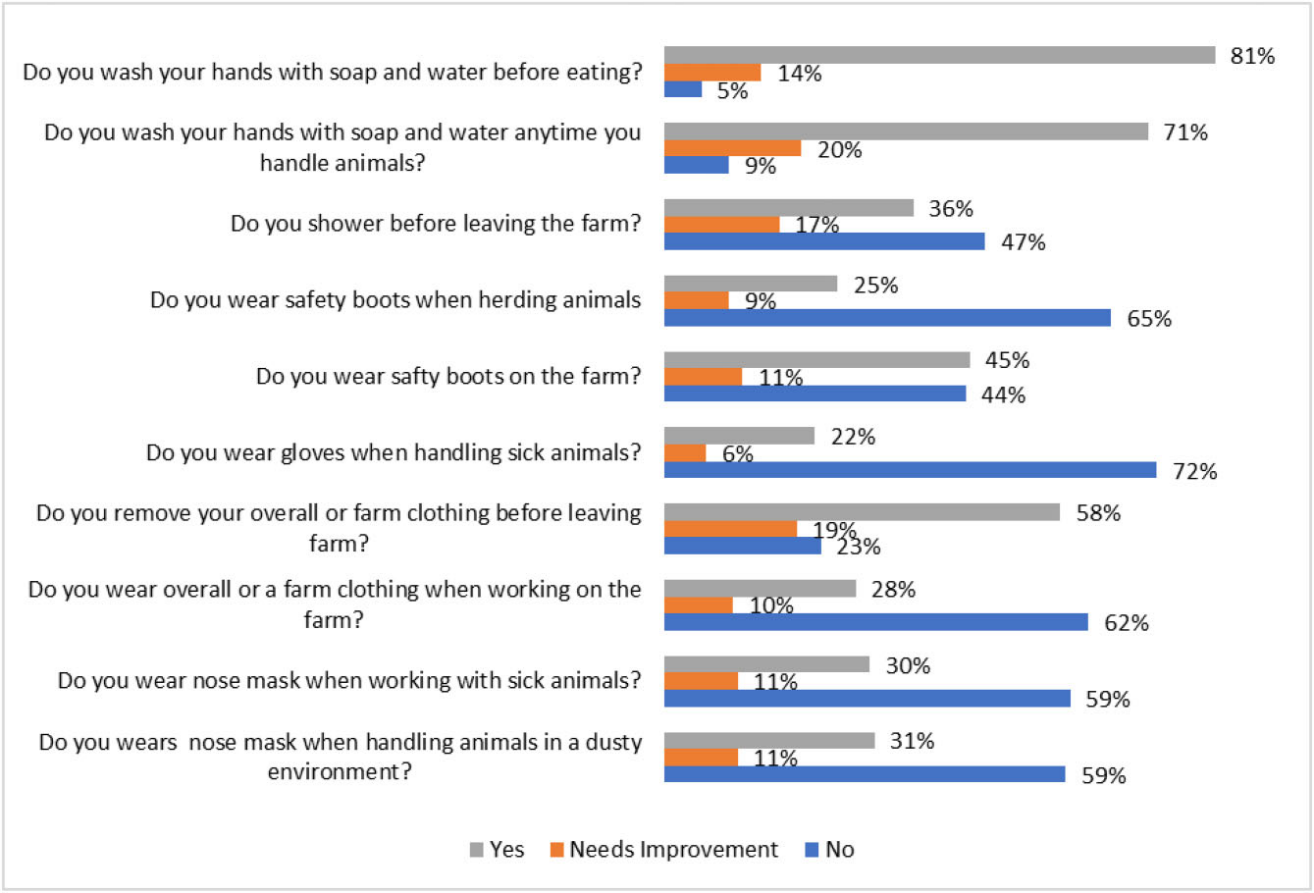
Farmers’ use of PPE and sanitary practices.

Figure 5 presents the proportion of farmers adopting PPE and sanitary practices. Most practices (7 of 10) were undertaken by less than half of the farmers (22–45%), primarily related to PPE use. In contrast, hand-washing practices were more common, with 70% and 80% of farmers adhering to these habits. Additionally, 58% of farmers who wore overalls reported removing them before leaving the farm. A significant proportion of farmers (6–20%) recognized the need for improvement in their practices.

### Farmers’ KAP scores

Table 2 presents the descriptive statistics for farmers’ total scores across five key domains. The mean scores for most domains were close to the maximum attainable score of 20, except for perception of disease transmission activities. The lowest mean score (8.6) was observed for the use of PPE and engagement in sanitary practices, indicating a gap in actual practice.

**Table 2.** Mean KAP scores.

Key domains	N	Minimum	Maximum	Mean	SD
Total number of diseases known by farmer	150	2	20	15.77	3.224
Total number of diseases perceived as zoonotic	150	5	20	13.47	3.510
Total number of PPEs and sanitary practices perceived as protective	150	0	20	16.03	6.282
Total number of PPE types and sanitary practices used	150	0	20	8.61	5.216

N: number of respondents.

Table 3 shows the proportion of farmers with poor or satisfactory scores in KAP domains. A majority (66%) demonstrated satisfactory knowledge of specific diseases, while about half had a satisfactory understanding of zoonotic diseases. Although 74% of farmers perceived the practices as protective, only 39% implemented these measures.

**Table 3.** Proportion of farmers having poor or satisfactory KAP scores.

Key domains	Poor, n (%)	Satisfactory, n (%)
Total number of disease types known	51 (34.0)	99 (66.0)
Total number of disease types perceived as zoonotic	76 (51.0)	73 (49.0)
Total number of practices perceived as protective	39 (26.0)	111 (74.0)
Total number of protective practices done	91 (60.7)	59 (39.3)

### Demographic and farm characteristic factors influencing farmers’ KAP

Table 4 presents the logistic regression analysis of satisfactory knowledge of specific disease types on various covariates. The Negelkerke R^2^ value of 0.32 indicates that the explanatory variables collectively account for 32% of the variation in satisfactory knowledge. Sex, production system and number of diseases encountered on the farm were significant predictors of knowledge level. Specifically, male farmers have three times higher odds of possessing satisfactory knowledge compared with female farmers (aOR 3.2, p<0.05). Farmers practicing intensive production systems have five times higher odds of having satisfactory knowledge compared with those with extensive systems (aOR 5.4). Conversely, an increase in the number of diseases encountered on the farm by one unit reduces the odds of possessing satisfactory knowledge by 50% (p<0.01).

**Table 4.** Association between socio-economic and farm characteristics and farmers’ knowledge of zoonotic diseases.

Characteristics	aOR	95% CI	p-Value
Sex (1=male, 0=female)	3.238	1.213 to 8.648	0.019
Production system in binary form (intensive=1, extensive=0)	5.396	1.276 to 22.808	0.022
Farm size in tropical livestock units	1.000	0.998 to 1.003	NS
Years in livestock farming (ref.: >10)			NS
<1	0.053	0.003 to 0.947	0.046
1–5	0.738	0.267 to 2.038	NS
6–10	1.743	0.577 to 5.267	NS
Level of education (ref.: tertiary education)			NS
No formal education	3.087	0.588 to 16.193	NS
Primary or junior high school	1.536	0.331 to 7.132	NS
Senior high school	2.823	0.580 to 13.739	NS
Number of diseases encountered on farm	0.499	0.347 to 0.717	0.000
Constant	1.006		0.995

NS: not significant; ref.: reference category.

### Demographic and farm characteristic factors influencing farmers’ attitude

Table 5 presents the logistic regression analysis of satisfactory perception of practices as protective on various covariates. The Negelkerke R^2^ value of 0.17 indicates that the covariates account for 17% of the variation in satisfactory perception. Being male negatively influences the odds of having satisfactory perception, with an aOR of 0.26, suggesting that males have 75% lower odds of perceiving practices as protective compared with females. Additionally, an increase in the number of diseases encountered on the farm by one unit reduces the odds of having satisfactory perception by 46%.

**Table 5.** Association between socio-economic and farm characteristics and practices perceived by farmer as protective.

Characteristics	aOR	95% CI	p-Value
Sex (1=male, 0=female)	0.261	0.077 to 0.885	0.031
Production system in binary form (intensive=1, extensive=0)	1.513	0.474 to 4.828	NS
Farm size in tropical livestock units	1.004	0.997 to 1.010	NS
Years in livestock farming (ref.: >10 )			NS
<1	0.652	0.046 to 9.330	NS
1–5	0.902	0.312 to 2.605	NS
6–10	0.762	0.267 to 2.176	NS
Level of education (ref.: tertiary)			NS
No formal education	2.658	0.542 to 13.024	NS
Primary or junior high school	1.610	0.397 to 6.525	NS
Senior high school	2.119	0.512 to 8.765	NS
Number of diseases encountered on farm	0.664	0.490 to 0.898	0.008
Constant	8.122		0.026

NS: not significant; ref.: reference category.

### Demographic and farm characteristic factors influencing farmers’ practices

Table 6 presents the logistic regression analysis of satisfactory performance of protective practices. The Negelkerke R^2^ value of 0.07 indicates that the covariates explain only 7% of the variation in satisfactory performance. The number of diseases encountered has a marginally significant negative effect (p<0.10), with each additional disease encountered on the farm associated with a 21% decrease in the odds of having a satisfactory perception of protective measures.

**Table 6.** Association between socio-economic and farm characteristics and farmers’ protective practices.

Characteristics	aOR	95% CI	p-value
Sex (1=male, 0=female)	1.666	0.671 to 4.134	NS
Production system in binary form (intensive=1, extensive=0)	1.042	0.392 to 2.765	NS
Farm size in tropical livestock units	1.000	0.999 to 1.002	NS
Years in livestock farming (ref.: >10)			NS
<1	1.620	0.192 to 13.672	NS
1–5	0.925	0.371 to 2.304	NS
6–10	1.056	0.436 to 2.560	NS
Level of education (ref.: tertiary)			NS
No formal education	1.269	0.314 to 5.131	NS
Primary or junior high school	1.154	0.311 to 4.280	NS
Senior high school	1.058	0.280 to 4.000	NS
Number of diseases encountered on farm	0.785	0.592 to 1.041	NS
Constant	0.539		NS

NS: not significant; ref.: reference category.

## Discussion

Zoonoses remain a major public health concern globally, especially in developing countries where close interactions between humans and animals are common and veterinary services are often limited. Despite their significance, there is a consistent gap in KAP concerning the prevention and control of zoonotic diseases, particularly among livestock farmers. This study examined KAP related to zoonoses among livestock farmers in Ghana, with a novel emphasis on multiple livestock species and varied production systems, expanding upon prior studies that focused primarily on single species or specific farming systems and a different geographical location.

The high awareness of disease transmission from livestock to humans among farmers in this study is consistent with previous studies, including research among cattle farmers in Tamale, Ghana,^17^ and livestock farmers in Vietnam.^24^ However, the lower awareness of the concept of reverse zoonoses (human-to-livestock disease transmission) indicates a knowledge gap that could impede effective disease control measures.

Awareness of specific zoonotic diseases varied among the farmers, with higher recognition for bird flu, rabies and swine flu and lower awareness for abortion-causing diseases and anthrax. The high awareness of avian influenza among Ghanaian farmers is consistent with previous studies among poultry farmers in Ghana^18^ but contrasts with the low awareness reported in the Vietnamese study,^24^ despite the disease being prevalent in both countries.^25,26^ The higher awareness of bird flu and rabies may be attributed to targeted public education efforts in the country, likely due to the significant impact of these diseases. In contrast, Ba et al.^24^ suggested that Vietnam faced information barriers, including limited accessibility to animal health workers and local authorities, as well as general and non-specific information from mass media.

The limited knowledge of abortion-causing diseases, such as brucellosis, aligns with findings by Abunna et al.,^27^ where most farmers were unaware of brucellosis as a zoonotic disease. This is understandable given the complex and often invisible symptoms of abortion-causing diseases like brucellosis compared with diseases with more tangible manifestations like ectoparasite infestations, worm infections, skin diseases and diarrhoeal diseases. Furthermore, anthrax's endemicity in northern Ghana may have contributed to the knowledge gap in the southern regions where the study was conducted.^15,28^ However, the awareness of swine flu might be influenced by its possible confusion with African Swine Fever, which is highly prevalent in Ghana.

Although most farmers perceived practices like handwashing and wearing PPE as protective, adoption of these practices was low, especially for wearing PPE. This finding is consistent with studies from Vietnam,^24^ Ghana,^17,18^ Nepal^29^ and Ethiopia.^30^ However, the relatively high use of the practice of handwashing agrees with findings by Ayim-Akonor et al.^18^ The limited use of PPE among farmers may be attributed to several factors, including financial constraints due to the small scale of most farms and risk-tolerant attitudes that lead farmers to underestimate the importance of protective gear. However, the specific factors contributing to low PPE use were not determined in this study. According to Ba et al.,^24^ farmers’ association of discomfort and inconvenience with the wearing of PPE may also play a role in their underutilization.

Education was not significantly associated with knowledge about zoonosis or the practice of protective measures, which contrasts with other studies that found a positive relationship between education and knowledge regarding zoonotic diseases^7,8,17,18,27,30^ and the practice of preventive measures.^8,30^ This discrepancy may be attributed to the limited education level of most farmers in the study, with the majority having no more than a basic (primary and junior high school) education. Community awareness programs and cultural norms may also play a role, warranting further investigation.

In this study, gender played a crucial role in farmers’ KAP related to zoonosis, with males having higher knowledge but a lower perception of protective practices. This paradox may be attributed to males’ overconfidence in their knowledge, leading to a lower perceived need for protection despite better disease understanding. Notably, this finding contrasts with previous studies in Ghana where gender was not a significant factor,^17,18^ perhaps due to differences in livestock management. Further research is needed to better understand these dynamics.

The number of diseases on a farm was negatively associated with farmers’ knowledge, perception and adoption of protective practices in this study. Limited knowledge about diseases and their prevention can hinder adoption of preventive measures, leading to increased disease infections. Farmers overwhelmed by disease burdens may prioritize immediate management over long-term prevention. Targeted interventions are therefore needed to bridge the gap between knowledge and practice and build capacity for sustainable disease prevention and management.

This study found that operators of intensive production systems have five times higher odds of knowing about specific zoonotic diseases compared with those in extensive systems, likely due to the organized nature of intensive farms. This finding aligns with Ayim-Akonor et al.,^18^ who noted that intensive farming systems are associated with higher disease awareness among poultry farmers and better management practices, often facilitated by formal training and regular veterinary services.

### Strengths and limitations

This study had several strengths and limitations. The strengths included a representative sample of livestock farmers, despite the potential limitation of sample size, and the effective use of images and videos to support data collection. Limitations included the lack of current livestock population data, which made determining the ideal sample size challenging, and the cross-sectional design, which limited tracking changes in KAP over time. However, the design was suitable for establishing a baseline. The use of visual aids helped mitigate challenges in describing specific diseases in local languages.

## Conclusions

Our study addresses the scarcity of data on awareness and practices among livestock farmers in developing countries. We found that despite satisfactory knowledge and attitudes towards zoonosis, practices related to prevention were unsatisfactory. Key factors influencing knowledge included gender, production system and diseases previously encountered, while gender and the number of diseases previously encountered on the farm impacted practices. To improve zoonoses prevention, we recommend targeted education on lesser-known zoonoses like anthrax and brucellosis, reverse zoonoses transmission and training for farmers in extensive systems. Further research on why women have lower odds of knowledge is also recommended.

## Data Availability

Data used for this study are available at https://data.mendeley.com/datasets/y5xzm79ysk/1.
